# The Effect of the Ultra-Marathon Run at a Distance of 100 Kilometers on the Concentration of Selected Adipokines in Adult Men

**DOI:** 10.3390/ijerph17124289

**Published:** 2020-06-16

**Authors:** Anna Czajkowska, Jadwiga Ambroszkiewicz, Anna Mróz, Katarzyna Witek, Dariusz Nowicki, Łukasz Małek

**Affiliations:** 1 Faculty of Physical Education, Józef Piłsudski, University of Physical Education, 00-968 Warsaw, Poland; anna.mroz@awf.edu.pl (A.M.); katarzyna.witek@awf.edu.pl (K.W.); dariusz.nowicki@awf.edu.pl (D.N.); 2 Department of Screening Tests and Metabolic Diagnostics, Institute of Mother and Child, 01-211 Warsaw, Poland; jadwiga.ambroszkiewicz@imid.med.pl; 3 Department of Epidemiology, Cardiovascular Disease Prevention and Health Promotion, National Institute of Cardiology, 04-628 Warsaw, Poland; lukasz.a.malek@gmail.com

**Keywords:** ultra-marathon, adipokine, resistin, chemerin, men

## Abstract

Pro-inflammatory adipokines have a multifunctional role in adipogenesis, angiogenesis, glucose homeostasis, and inflammation. The aim of the present study is to evaluate the effect of running a 100 km ultra-marathon on serum levels of two adipokines: resistin and chemerin. Fifteen male participants complete a medical questionnaire and their body composition is assessed. Serum resistin, chemerin, high sensitivity C-reactive protein (hs-CRP), glucose, and lactate levels are measured at baseline and post-race. During-race data on fluid and food consumption and energy expenditure are calculated. There is a higher (*p* < 0.001) post-race concentration of resistin and hs-CRP compared with resting values, with no change in chemerin levels. There is an inverse correlation of the change in resistin levels with post-run glucose values (*r* = 0.742, *p* < 0.001) and a positive correlation between changes in hs-CRP and energy expenditure (*r* = 0.782, *p* < 0.001). The present results show the impact of running an ultra-marathon on serum levels of pro-inflammatory markers released by adipose tissue. It is difficult to establish whether these results may be due to the stress of exercise, high energy expenditure or caloric deficit. However, we suggest that an addition of resistin to traditional pro-inflammatory markers (including CRP) may improve the assessment of inflammation in conditions of high-energy expenditure.

## 1. Introduction

Adipose tissue produces multiple cytokines, including pro-inflammatory as well as anti-inflammatory factors, which may modulate inflammatory processes, insulin sensitivity, endothelial function, and atherosclerosis [1]. Added to well-known adipokines, such as leptin and adiponectin, resistin and chemerin may be of interest to researchers and, after establishing reference values, also for clinicians due to their multifunctional role in adipogenesis, angiogenesis, glucose homeostasis, and inflammation [2,3,4,5,6].

Resistin is a 12.5 kDa cysteine-rich protein up-regulated during adipocyte differentiation and down-regulated in mature adipocytes. Human resistin consists of 108 amino acids and is a disulfide-linked homodimer circulating in blood as a dimeric protein of two 92-amino-acid polypeptides. Resistin also can dimerize as heterodimers, through a disulfide bond. Its production is regulated by various factors, depending on cell type. It is synthesized not only in adipose tissue, but also in macrophages [2,3,7]. Therefore, resistin has pro-inflammatory properties and its higher levels indicate the development of insulin resistance, diabetes, obesity, and cardiovascular disease. [8,9,10,11,12,13], however, the exact mechanism is not clear. The results of previous studies suggest that resistin has various regulatory effects on energy metabolism and thermogenesis [14].

A study assessing the relationship between the serum concentration of resistin and physical activity was conducted on a large adult population by Marcelino et al., [15]. The authors observed that resistin was inversely correlated with measures of physical activity, making this protein a potentially useful biomarker of physical activity. Other studies point out that resistin changed, dependent on exercise intensity and volume. No changes in serum resistin levels were observed in submaximal efforts [16] or in relation to the type of physical activity [17]. However, post-exercise increases in resistin concentration were observed in conditions of significant energy deficits such as running a marathon [18] or ultra-marathon [19]. Moreover, at the end of the recovery phase (20 h) serum resistin levels were reduced compared to post-exercise levels but remained significantly elevated compared to pre-race values [19].

Another important newly discovered adipokine, chemerin, is released from the cells (most abundant in hepatocytes and adipocytes) as a biologic inactive prochemerin, which is activated by C-terminal proteolysis [4,5]. Extracellular cysteine and serine proteases generate different isoforms of chemerin with chemerin 157 being the most active form [6,20]. Chemerin acts through its receptors: chemokine-like receptor 1 (CMKLR1), G-protein couplet receptor 1 (GPR1) and C-C chemokine receptor-like 2 (CCRL2) and plays a critical role in metabolic and inflammatory activities [21,22]. This adipokine can regulate adipocyte differentiation and also can stimulate chemotaxis of macrophages and dendritic cells leading to inflammatory activities. Dependent on the context (different signaling pathways), chemerin may act as a pro-inflammatory or anti-inflammatory mediator [23,24]. Elevated circulating chemerin is associated with inflammation, metabolic syndrome, and obesity. There is a significant and positive correlation between chemerin level and body mass index, waist–hip ratio, waist circumference or visceral adipose tissue mass [6,25]. Serum chemerin concentration was significantly reduced after different interventions to reduce fat mass: 12 weeks of exercise, a six month calorie-restricted diet, and bariatric surgery [26]. Similarly, a study by Faramarzi et al., [27] showed that aerobic exercise in overweight women caused a reduction in body fat and a significant decrease in serum chemerin levels. There are no studies about chemerin function in continuous, prolonged bouts of moderate-intensity physical exercise such as running an ultra-marathon. We speculate that chemerin, with its pleiotropic biological properties, seems to be an interesting biomarker in the assessment of inflammation in prolonged aerobic exercise. It is shown to regulate insulin-stimulated glucose uptake in skeletal muscle [28]. It remains unclear whether chemerin is associated with chronic heart failure [29,30], renal dysfunction [31], and cancer [32,33]

The impact of physical activity on other post-inflammatory indicators, such as C-reactive protein (CRP) level, is well described in the literature [34]. Many authors also noticed the relationship between pro-inflammatory factors and energy balance when assessing the amount of energy spent on effort with the energy supplied in the form of food, which may be an important element of pro-inflammatory reaction during exercise [19,35].

The aim of the present study is to evaluate the effect of continuous, prolonged, moderate-intensity running exercise, such as running a 100 km ultra-marathon, and acute energy deficit on serum levels of pro-inflammatory adipokines: resistin and chemerin. Moreover, the study attempts to assess the relationship of these adipokines with parameters, such as: CRP, glucose, lactate, energy intake, and energy expenditure measured pre- and post-race.

## 2. Materials and Methods

### 2.1. Participants

The study was conducted during a 100 km running ultra-marathon on a flat terrain (asphalt bitumen track and short parts of cobblestone), which took place on 10 November 2018 at the University of Physical Education in Warsaw (www.supermaraton100lecia.pl). The race consisted of 65.10 laps of 1535.89 m long and was accredited by the Polish Athletics Association as National Championships on 100 km.

The study was approved by the Ethics Committee of the Regional Medical Chamber in Warsaw (number 52/17), with written informed consent obtained from all participants.

Taken from 204 runners taking part in the race, we included 15 men—amateur, healthy runners—who volunteered to participate in the study and were obliged to follow the whole protocol of the study. Right before the start of the race each participant underwent screening in the form of a medical questionnaire to exclude any known medical conditions. Assessment of body composition, high sensitivity C-reactive protein (hs-CRP) and adipokines (resistin and chemerin) were done. Concurrently, analysis of baseline capillary lactic acid (LA) and glucose (Glu) concentration was performed. 

During the race, after each lap, we collected data on fluid and food intake. Estimation of caloric content from the completed forms was based on information provided in the “Food and Nutrition Tables” [36]. Fluids drank were summed. Immediately after the end of running, each participant underwent a final assessment of capillary LA and Glu concentration, serum resistin, and chemerin, as well as hs-CRP concentrations. Collaborating with a certified company (datasport.pl), we collected data on duration of the race, mean pace and total distance covered by each runner participating in the study. 

Body composition was analyzed using a Tanita BC 41 MA (Tanita Inc., Tokyo, Japan) device. A cardiopulmonary exercise test (CPET) on a treadmill (Saturn, h/p/cosmos, Nussdorf–Traunstein, Germany) was performed within 4 weeks of the race. Using the portable metabolic system (Metamax 3B, Cortex Biophysik GmbH, Leipzig, Germany), oxygen consumption (VO2) was measured. A progressive exercise test was performed with the treadmill speed increased every 2 min. by 1 km/h. Data from the individual running speed tests and oxygen uptake were used to calculate the running energy cost of the race using the indirect calorimetry method [37]. The amount of energy spent per run was compared with the amount of energy provided in the form of food. The energy balance was presented as a percentage of the ratio of calories provided in the form of food to the energy expenditure on effort.

### 2.2. Biochemical Analysis

Capillary lactic acid (LA) and glucose (Glu) assessments were performed using the Biosen C-Line Glucose and Lactate analyser (EKF Diagnostics, Cardiff, United Kingdom). To assess high sensitivity C-reactive protein (hs-CRP) levels, an electrochemiluminescence immunoassay method (ECLIA) using a Roche Cobas e411 analyzer (Roche Diagnostics, Mannheim, Germany) was used. The detection limit in this method was 0.3 mg/L.

Serum samples were obtained and frozen at −20 °C until analysis of adipokines using a commercial enzyme-linked immunosorbent assay (ELISA), according to the manufacturer’s instructions. All samples were tested in duplicate. Serum resistin and chemerin levels were determined using kits from Mediagnost (Reutlingen, Germany). Concerning resistin, the intra-assay variation coefficients (CV) ranged between 4.49–4.97%, and inter-assay CV between 3.37–6.67%. Regarding chemerin, the intra-assay CV was between 2.02–2.76%, and the inter-assay CV was between 4.38–5.87%. The analytical sensitivity of these assays was 12 pg/mL for resistin and 5 pg/mL for chemerin.

### 2.3. Statistical Analysis

All results for categorical variables were presented as a number. Continuous variables were expressed as mean values and standard deviations (SD) or median values and interquartile ranges (IQR) according to normal or non-normal data distribution. A Wilcoxon test for paired samples or a Mann–Whitney test for unpaired samples were applied to compare continuous data. To assess the correlation between continuous variables, the Spearman test was applied. All tests were two-sided with a significance level of *p* < 0.05. Statistical analyses were performed using Statistica 10 PL (Kraków, StatSoft, Poland).

## 3. Results

Anthropometric and running characteristics at baseline and training history of studied participants are expressed in Table 1 and Table 2.

**Figure 1:**
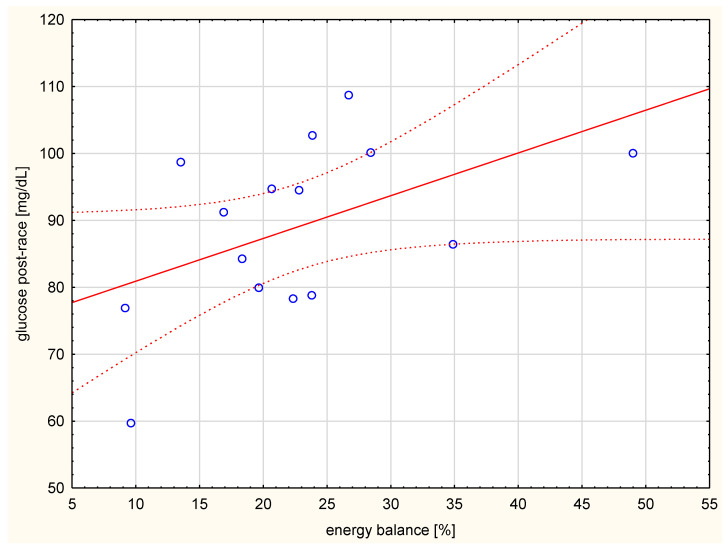
Correlation between energy balance (%) and glucose level after the run (*r* = 0.589, *p* < 0.05).

The subjects were not high-performance athletes and not all of them completed the whole 100 km distance. They did not differ significantly in their developed running speed. The effort was at a mean level of 57.2 ± 3.5% maximal oxygen consumption (VO_2max_). The average caloric intake for these participants was 1500.0 ± 918.4 kcal/race. Their corresponding average energy expenditure was 7167.0 ± 1271.8 kcal, indicating a negative caloric balance. The value of the energy balance was significantly correlated with the glucose level after the run (Figure 1).

The results of pre-race and post-race biochemical evaluation are presented in Table 3. We did not observe differences in serum chemerin, glucose, and lactic acid levels at baseline and after the run. However, we found significantly higher (*p* < 0.001) post-race concentrations of resistin and hs-CRP compared with resting values.

None of the adipokines showed a correlation with body mass index (BMI) and fat. The relationships of pre- and post-run chemerin concentrations with resistin and hs-CRP levels are shown in Table 4. The pre-run chemerin level was significantly and directly correlated with pre-run resistin and pre-run hs-CRP levels, however, post-run chemerin was significantly associated with post-run resistin levels.

**Figure 2:**
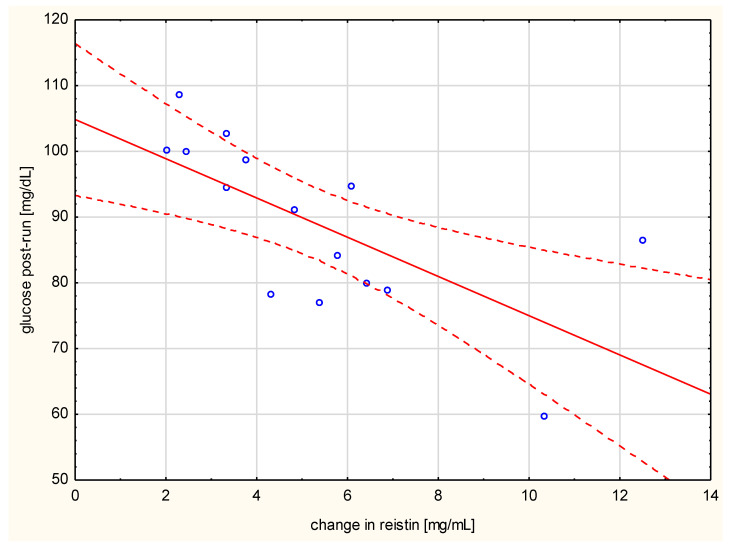
Correlation of change in resistin levels with post-run glucose values (*r* = −0.742, *p* < 0.001).

**Figure 3:**
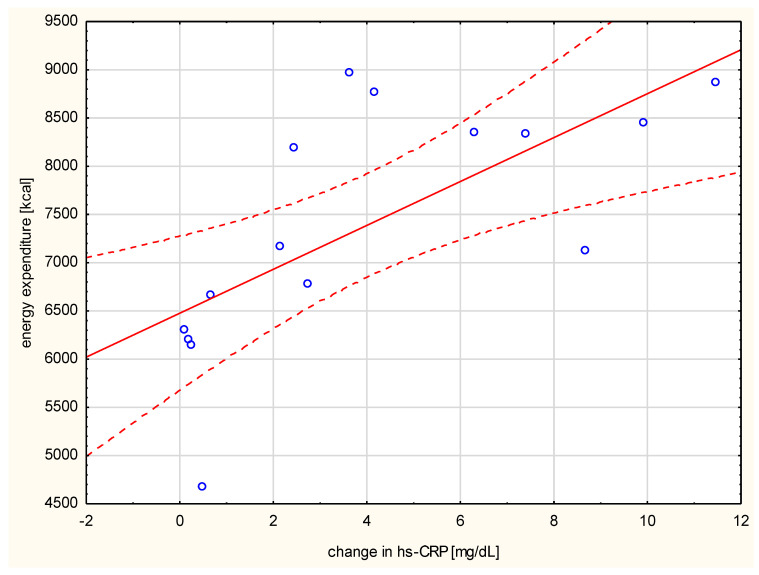
Correlation between the run energy expenditure and the change in serum hs-CRP (*r* = 0.782, *p* < 0.001).

Negative correlation of the changes in resistin levels with post-run glucose values (*r* = 0.742, *p* < 0.001, Figure 2) was observed. The change in hs-CRP correlated with the distance run (*r* = 0.521, *p* < 0.05), the duration of the run (*r* = 0.876, *p* < 0.01), and the amount of energy spent per run (*r* = 0.782, *p* < 0.001) (Figure 3).

## 4. Discussion

Running an ultra-marathon is an exhausting effort for the whole organism. Prolonged aerobic exercise, aside from the benefits of physical activity on the metabolism, leads to a pro-inflammatory profile [16,17]. The findings of the present study showed that members of an ultra-marathon had elevated serum resistin and high sensitivity C-reactive protein (hs-CRP) levels compared to their resting values. However, no changes were recorded for chemerin concentrations. To our knowledge, this is the first study to investigate the effect of running an ultra-marathon on circulating chemerin. 

Some authors observed increased levels of resistin in athletes who ran a variety of distances [18,19,38]. They detected increased serum resistin levels in the middle-distance and marathon runners, but not in the sprinters, compared among lean young individuals. Roupas et al., [19] examined serum adipokine concentrations in 17 male athletes participating in a 180 km ultra-marathon race. Upon conclusion of the race, they observed significantly (*p* < 0.001) higher resistin levels and reduced leptin levels compared with baseline values. They found no significant changes in the serum concentration of other adipokines, such as adiponectin and visfatin. The authors suggested that prolonged aerobic exercise and an acute negative energy balance (a deficit of about 5000 kcal) lead to up-regulation of circulating resistin levels and down-regulation of serum leptin levels. Vuolteenaho et al., [18] assessed leptin, adiponectin, and resistin levels in 46 male marathoners. They reported significantly increased resistin (by 107%) and adiponectin (by 13%) levels immediately after the marathon race, but no change in the leptin concentrations. Sansoni et al., [39], considering 17 mountain ultra-marathoners (65 km), observed significantly decreased leptin levels (*p* < 0.001) and significantly (*p* < 0.001) increased levels of resistin and visfatin post-run.

Increased resistin expression has been correlated to inflammatory markers, such as interleukin-6 and tumor necrosis factor α (TNF-α) [40]. Since it is known that transmembrane lipoprotein receptor 4 (TLR4) is a receptor for resistin, pro-inflammatory properties of resistin are mediated through the nuclear factor ĸ-light-chain-enhancer of activated B cells (NFĸB) and the mitogen-activated protein kinase (MAPK) signaling mechanisms [41]. Moreover, some authors suggested that resistin is not only a pro-inflammatory indicator, but also an energy deficit signaler, and is related to insulin resistance [19]. Literature data suggested the effect of chronic energy restriction and eating disorders on resistin levels [42,43].

Here, the energy balance was similar to that in other reports [44,45]. Prolonged exercise usually caused energetic deficits through insufficient energy supply in the form of food during the race. However, there were no changes in glucose levels related to food intake. Ishihara et al., showed that food intake did not influence glucose concentration, but positively correlated with running speed [45]. During our study, we observed a significant positive correlation between post-run glucose level and energy balance, and a significant negative relationship between changes in resistin and post-run glucose. This suggests that resistin may influence the maintenance of glucose homeostasis. 

An average glucose concentration from the start to the end was relatively stable during our study. Other authors had similar observations [46,47]. Occasionally serum concentrations of glucose during an ultra-marathon run changed. Those situations included races when uniform nutrition with the use of high energy gels was provided, leading to higher glucose values after the run. However, if subjects were fed in whichever manner, an increase in glucose concentration was not observed. Hansen et al., [48] suggested that a higher total carbohydrate intake during the race gave the opportunity for glucose synthesis in the post-completion phase. During our study, each participant consumed food prepared by himself, and a large variety of nutrition models were observed. There were also large discrepancies between the energy balances of individual subjects.

The observed changes in CRP levels after prolonged efforts differed when comparing the marathon and ultra-marathon races. Wołyniec et al., [45] reported that the increase in CRP after the run was eight times higher in the ultra-marathon than after the marathon. Recently, many authors have described the relationship between various forms and the intensity and volume of physical activity with the level of CRP. Exercise causes a short-term inflammatory response and has a long-term “anti-inflammatory” effect. This anti-inflammatory response can contribute to the beneficial effects of habitual physical activity [34]. Similar to our results, an increase in hs-CRP concentration was observed in other studies after 75 and 100 km races. These changes lasted up to 14 h post-race [46,49].

This study has some limitations. First, it is a field study that carries all the limitations of similar studies, such as a limited number of registered athletes, so as not disturb exhausted athletes with additional interventions. We did not measure other inflammatory factors such as interleukins and TNFα. Among adipokines, we determined resistin and chemerin but did not examine levels of anti-inflammatory adipokines such as adiponectin and omentin. Noteworthy, we assessed chemerin levels in ultra-marathon runners for the first time. However, we did not observe differences in serum chemerin levels before and after the run. It is difficult to say whether the stress of exercise itself, or the high energy expenditure/caloric deficit, or all these things combined influenced our results regarding pro-inflammatory markers. Another limitation is that we did not measure levels of pro-inflammatory markers, including adipokines, at 24–72 h post ultra-marathon. Therefore, we did not observe if their values return to normal after the run.

## 5. Conclusions

The present results showed the impact of running an ultra-marathon on adipokine levels released from adipose tissue leading to the trend of a pro-inflammatory profile. The addition of resistin to traditional pro-inflammatory markers (including C-reactive protein) may improve the assessment of inflammation in conditions of high-energy expenditure. However, this study was conducted just in men so cannot be generalized to everyone. Further studies are needed to clarify whether resistin deeply contributes to the ultra-marathon-related inflammatory status and might be a potential novel biomarker.

## Figures and Tables

**Table 1:** Anthropometric and training characteristics at baseline and training history of subjects.

Parameter	Mean Value ± SD or Median Value and IQR
Age (years)	42.5 ± 8.9 ^a^
Body weight (kg)	78.2 ± 8.8 ^a^
Height (cm)	178.0 ± 4.3 ^a^
Body fat (%)	13.3 ± 3.2 ^a^
Body fat free mass (kg)	67.6 ± 5.9 ^a^
BMI (kg/m^2)^	24.6 ± 2.2 ^a^
Duration of training (years)	5.0 (3.5–6.0) ^b^
Weekly running distance (km)	70 (45–85) ^b^

Data were expressed as ^a^ mean values ± standard deviation (SD) or ^b^ median values and interquartile ranges (IQR) according to normal or non-normal data distribution; BMI—body mass index.

**Table 2:** Ultra-marathon 100 km race performance measures.

Parameter	Median and IQR
Distance (km)	90.8 (70.8–100.0)
Ultra-marathon time (h)	10.0 (8.3–11.5)
Ultra-marathon running velocity (km/h)	8.7 (8.5–9.7)
Energy intake during the run (kcal)	1500 (1130–2026)
Liquid intake during the run (ml)	1850 (1300–2500)
Energy expenditure (kcal)	7167 (6266–8443)
Energy balance (%)	22.65 (16.92–26.70)

Data are expressed as median values and interquartile ranges (IQR).

**Table 3:** Biochemical parameters in the serum of participants at baseline and post-race.

Parameter	Pre-Race	Post-Race	*p*
hs-CRP (mg/L)	0.52 (0.43–1.11)	3.23 (1.87–8.11)	0.0006
Resistin (ng/mL)	5.26 (4.42–5.98)	9.51 (8.60–12.64)	0.0006
Chemerin (ng/mL)	91.61 (85.17–104.45)	87.15 (78.24–102.53)	0.394
Glucose (mg/dL)	88.0 (82.60–102.70)	81.20 (78.80–100.00)	0.649
Lactic acid (mmol/L)	1.81 (1.71–2.40)	2.22 (1.36–4.57)	0.099

Data were expressed as median values and interquartile ranges (IQR); hs-CRP—high sensitivity C-reactive protein.

**Table 4:** Spearman coefficients correlation of pre- and post-run chemerin levels with levels of resistin and hs-CRP.

	Chemerin Pre-Run		Chemerin Post-Run	
	*r*	*p*	*r*	*p*
Resistin pre-run	0.617	<0.05	0.198	NS
Resistin post-run	0.367	NS	0.620	<0.05
hs-CRP pre-run	0.578	<0.05	0.061	NS
hs-CRP post-run	0.077	NS	−0.334	NS

hs-CRP—high sensitivity C-reactive protein.
